# Personalized neoantigen cancer vaccines: current progression, challenges and a bright future

**DOI:** 10.1007/s10238-024-01436-7

**Published:** 2024-09-26

**Authors:** Da-Wei Wu, Shuo-Peng Jia, Shu-Jun Xing, Hai-lan Ma, Xin Wang, Qi-Yu Tang, Zi-Wei Li, Qing Wu, Min Bai, Xin-Yong Zhang, Xiao-Feng Fu, Ming-Ming Jia, Yu Tang, Li Chen, Ning Li

**Affiliations:** 1 Clinical Trials Center, National Cancer Center/National Clinical Research Center for Cancer/Cancer Hospital, Chinese Academy of Medical Sciences and Peking Union Medical College, Beijing, 100021 China; 2 Clinical Trials Center, Shanxi Province Cancer Hospital/Shanxi Hospital Affiliated to Cancer Hospital, Chinese Academy of Medical Sciences/Cancer Hospital Affiliated to Shanxi Medical University, Taiyuan, 030013 China; 3 Department of Hematology, Shanxi Province Cancer Hospital/Shanxi Hospital Affiliated to Cancer Hospital, Chinese Academy of Medical Sciences/Cancer Hospital Affiliated to Shanxi Medical University, Taiyuan, 030013 China; 4 Department of Basic Medicine and Clinical Pharmacy, China Pharmaceutical University, Nanjing, 211198 China; 5 Department of Medical Oncology, Beijing Chest Hospital of Capital Medical University, Beijing, 100021 China; 6 Beijing Likang Life Science, Beijing, 100000 China

**Keywords:** Neoantigen vaccines, Solid tumor, Personalized, Clinical trial

## Abstract

Tumor neoantigens possess specific immunogenicity and personalized therapeutic vaccines based on neoantigens which have shown promising results in some clinical trials, with broad application prospects. However, the field is developing rapidly and there are currently few relevant review articles. Summarizing and analyzing the status of global personalized neoantigen vaccine clinical trials will provide important data for all stakeholders in drug development. Based on the Trialtrove database, a retrospective analysis was conducted using trial quantity as a key indicator for neo-adjuvant and adjuvant therapy anti-PD-1/PD-L1 clinical trials initiated before the end of 2022. The time trend of newly initiated trials was investigated. The sponsor type, host country, treatment mode, combination strategy, tested drugs, and targeted cancer types of these trials were summarized. As of December 2022, a total of 199 trials were included in the analysis. Among these studies, Phase I studies were the most numerous (119, 59.8%), and Phase I studies have been the predominant study type since 2015. Peptide vaccines were the largest neoantigen vaccines type, accounting for 64.8% of all clinical trials. Based on peptide delivery platforms, the proportion of trials was highest for the DC system (32, 16.1%), followed by LNP (11, 5.5%), LPX (11, 5.5%), and viruses (7, 3.5%). Most vaccines were applied in trials as a monotherapy (133/199, 66.8%), meanwhile combining immunotherapeutic drugs was the most common form for combination therapy. In terms of indications, the largest number of trials involved three or more unspecified solid tumors (50/199, 25.1%), followed by non-small cell lung cancer (24/199, 12.1%) and pancreatic cancer (15/199, 7.5%). The clinical development of personalized neoantigen cancer vaccines is still in the early stage. A clear shift in delivery systems from peptides to DC and liposomal platforms, with the largest number of studies in Asia, collectively marks a new era in the field. The adjuvant or maintenance therapy, and the combination treatment with ICIs are becoming the important clinical development orientation. As research on tumor–immune interactions intensifies, the design, development, and application of neoantigen vaccines are bound to develop rapidly, which will bring a new revolution in the future cancer treatment.

## Introduction

Tumor immunotherapy is a treatment approach based on the human immune system, aimed at treating cancer by activating and regulating the immune system [1]. In recent years, immunotherapy has developed rapidly, providing a novel treatment approach beyond surgery, conventional chemotherapy, and radiation therapy, opening up a new era in cancer treatment. Immune checkpoint inhibitors, as agents targeting programmed cell death protein 1 (PD-1), programmed death-ligand 1 (PD-L1), cytotoxic T lymphocyte antigen 4 (CTLA-4), lymphocyte activation gene 3 (LAG-3), T cell immunoglobulin domain and mucin domain 3 (TIM-3) and others, are the hot research directions of immunotherapy in recent years. After having demonstrated clinical benefit in multiple solid tumors and lymphoma as a monotherapy or combinations with standard of care, the trends in terms of novel combinations, such as with cancer vaccine, or exploring biomarkers for predicting of response/resistance/irAE [2–6] have risen above the horizon.

Along with the large-scale clinical application of mRNA technology, cancer vaccines have gradually become a research hotspot after immune checkpoint inhibitors. Tumor vaccines are designed to enhance antigen presentation, activate antigen-specific effector function, and induce memory T cell-mediated killing, thereby exerting their immunotherapeutic effects [7]. Traditional cancer vaccines designed to target tumor-associated antigens (TAAs) have limited success due to poor tumor specificity [8].

Tumor-specific antigens (TSAs), also known as neoantigens, are self-antigens produced by tumor cells due to genomic mutations. They originate from non-synonymous mutations and other genetic changes in cancer cells, such as genomic alterations, RNA splicing dysregulation, disordered post-translational modifications, and open reading frames (ORFs) produced by viral coding[9]. Compared to traditional vaccines, neoantigen vaccines have several advantages: (1) They can effectively stimulate, enhance, and diversify anti-tumor T cell responses, maximizing therapeutic specificity and overcoming immune tolerance [10]; (2) its strong affinity with major histocompatibility complex (MHC) molecules can prevent immune cells from attacking normal cells of patients and ensure the safety of treatment [11]; and (3) they are highly feasible, generally safe, and easier to manufacture.

Currently, personalized tumor neoantigen vaccines have made significant progress in clinical trials and have shown promising results in treating various cancers. Research has shown that over 60% of melanoma patients who received personalized tumor neoantigen vaccines remained recurrence-free or did not progress after two years [12]. In recent years, personalized neoantigen vaccines have continued to make progress in combination with other immunotherapies. Studies have shown that combining it with immune checkpoint blockade (ICB) can significantly improve the survival rate of melanoma patients [13]. For advanced non-squamous non-small cell lung cancer, personalized neoantigen vaccines combined with chemotherapy and anti-programmed cell death protein 1 (PD-1) therapy have strong effects, especially on CD4 T cells[14]. Tumor neoantigen vaccines, as a personalized treatment method, have enormous potential in the field of cancer treatment, but more research and development is still needed to improve their efficacy and application.

There is ample reason to believe that neoantigen-based therapy will become a promising field of cancer immunotherapy. This article summarizes and discusses the clinical trial overview and latest developments of personalized cancer vaccines based on neoantigens as of December 2022, thus identifying challenges and future opportunities.

## Materials and methods

Clinical trial data were sourced from the Trialtrove database, which comprehensively tracks the R&D pipeline from preclinical to market globally, covering a wide range of information such as drug, company, development history or regulatory status in detail. This article was searched using personalized oncology and neoantigen oncology as keywords. As of 31 December 2022, 220 oncology clinical trials of personalized neoantigens were retrieved from this database. Personalized neoantigen clinical trials were then identified through manual review by two independent oncologists. In the event of disagreement, a third-party oncologist was invited to arbitrate until a unified decision was made. As adaptive cell therapies such as tumor infiltrating lymphocytes (TILs), T cell receptor engineered T cells (TCR-T) and chimeric antigen receptor T cells (CAR-T) are not typical cancer vaccines and were excluded and there were 199 trials eligible for this study.

**Fig. 1:**
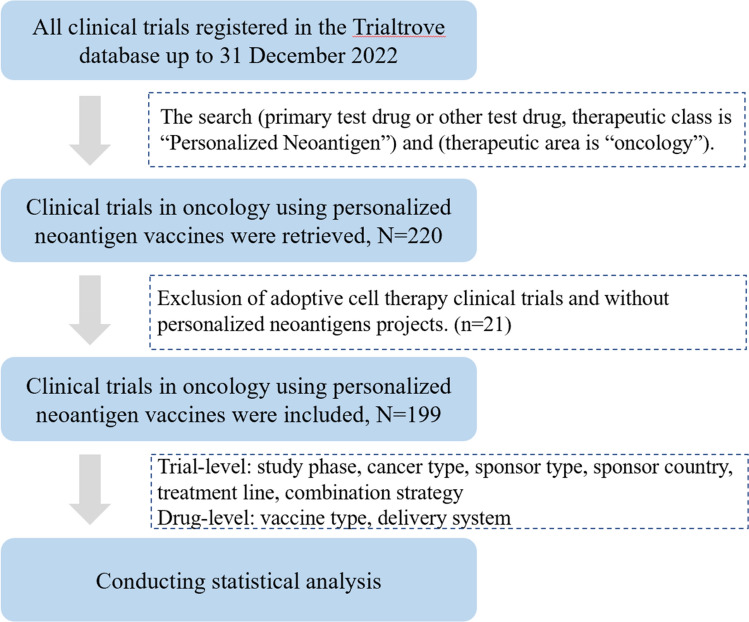
Data processing flow and variables

The analysis was conducted in the following manner: (i) the number of new trials initiated up to December 2022; (ii) the number of trials by sponsor type and host country; (iii) the number of trials by treatment modality, combination strategy and test drug; (iv) the number of trials by cancer type for different time periods; and (v) uniformly labeled as an unspecified solid tumor if multiple tumor indications are available (Fig. 1).

## Results

**Fig. 2:**
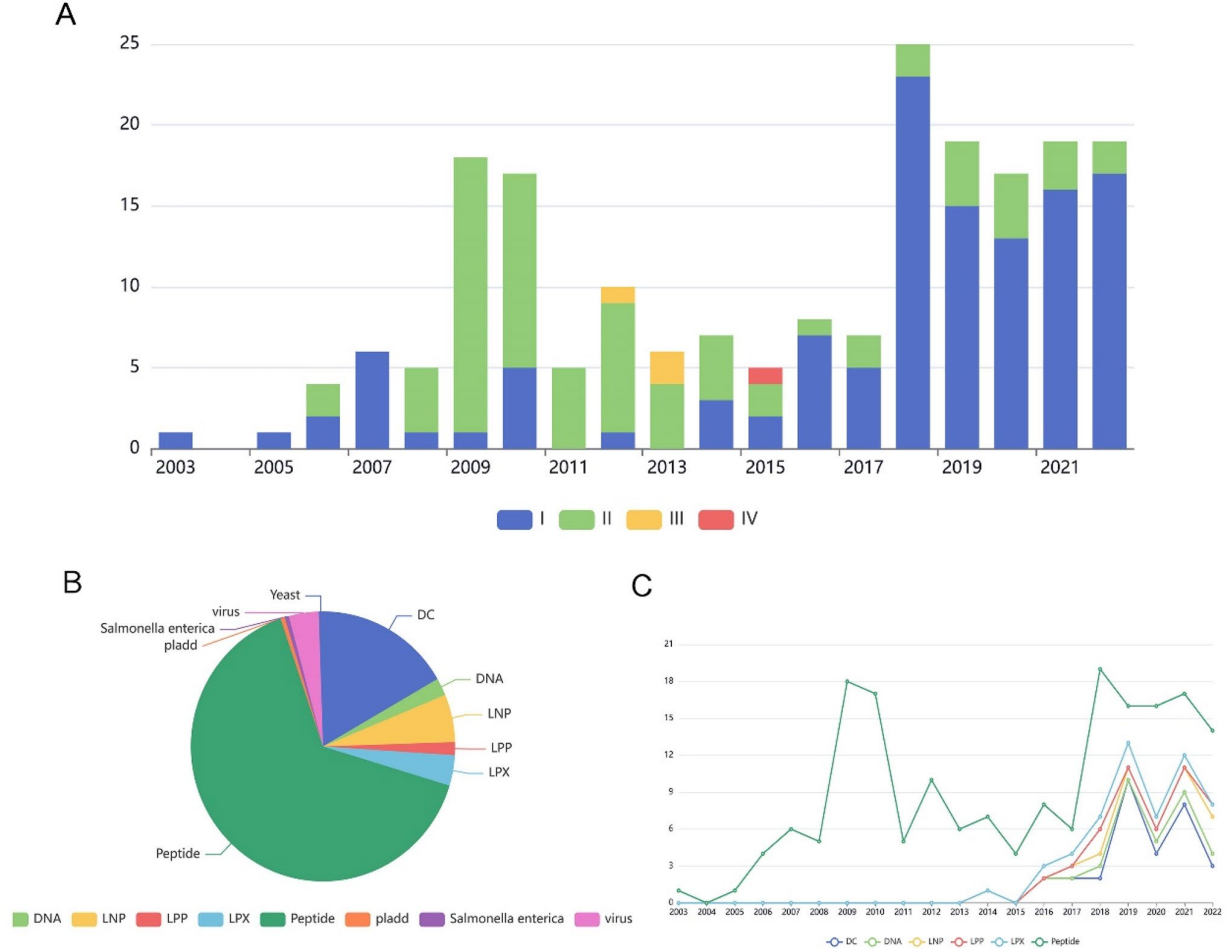
**A** The number of clinical trials of personalized neoantigen vaccines registered each year on the Trialtrove database. **B** Nonantigenic vaccine loading platforms and temporal trends, PPV, DC, mRNA

As shown in Fig. 2A a total of 199 clinical trials of personalized neoantigen oncology vaccines were initiated globally from 2003 to December 2022. The number of clinical trials has generally shown an increasing trend, with two peaks in 2009 and the number of clinical trials in 2022 is the same as in 2021, as some trials had not yet started at the time of writing. In terms of trial phases, Phase I studies had the highest number (119, 59.8%), followed by Phase II studies (76, 38.2%), with Phase III studies (3, 1.5%) and Phase IV studies (1, 0.5%) being less common. Prior to 2015, Phase II clinical studies were mainly conducted, while after 2015, Phase I studies became more prevalent.

As shown in Fig. 2B and C, the delivery systems of the 199 personalized neoantigen vaccines against solid tumors involved in this study were analyzed, with the largest proportion of peptide-based delivery platforms (125, 62.9%), followed by dendritic cells (DC) systems (32, 16.1%), lipid nanoparticle (LNP) (11, 5.5%), lipoplexes (LPX)(11, 5.5%) and virus (7, 3.5%). Observing the evolution of delivery systems over time, from 2003 to 2015, peptides were the main platform for new drug testing, and since 2015, delivery systems such as DC, DNA, LNP, LPX and lipopolyplex (LPP) have started to grow rapidly.

**Fig. 3:**
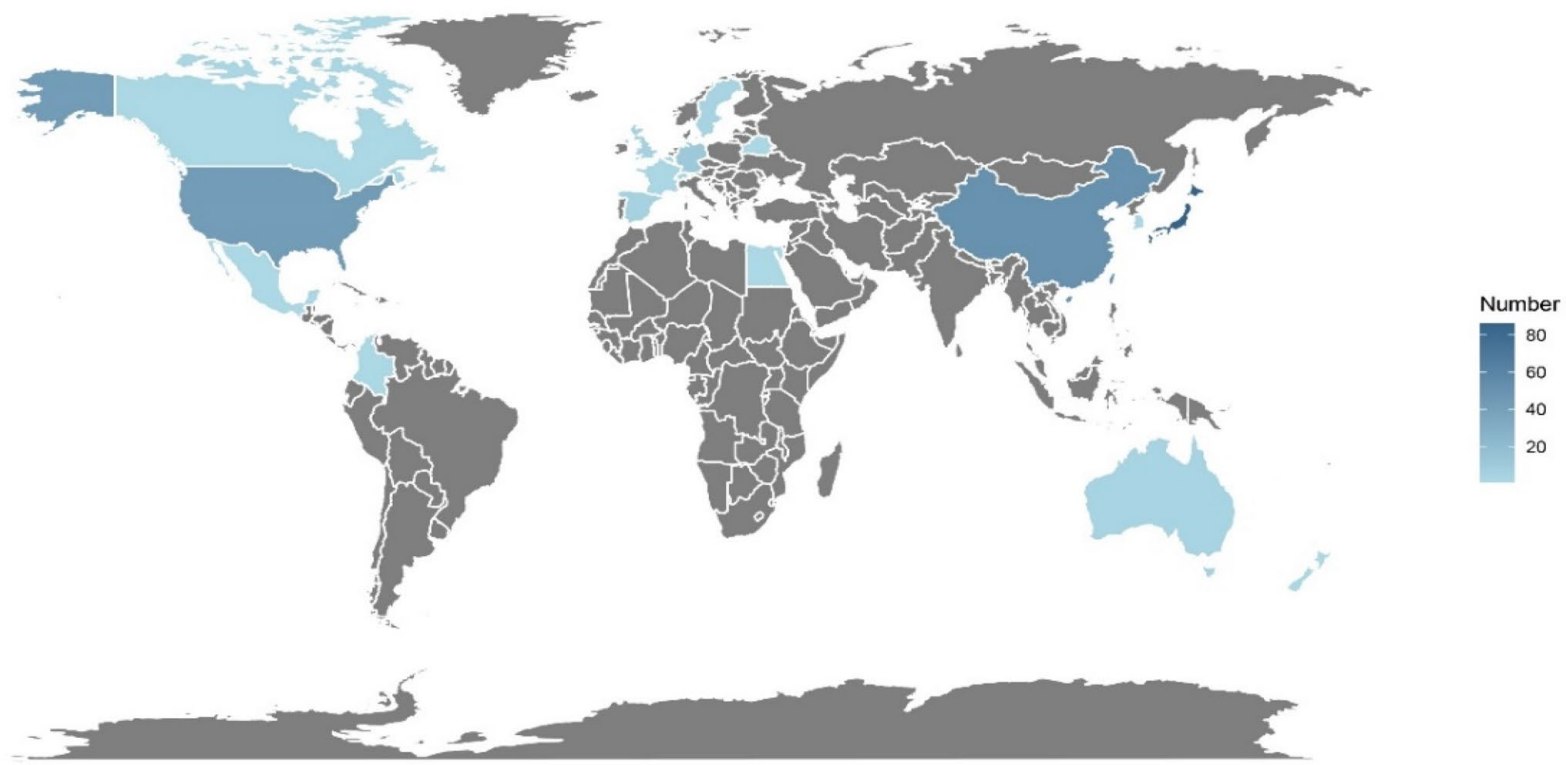
An overview of personalized neoantigen vaccine clinical trials in global

As some of the trials are international multi-center studies, therefore there are multiple countries participating together. Figure 3 shows an overview of global clinical trials of personalized neoantigen vaccines. The participating countries in 140 (62.8%) trials were in Asia, 45 (20.2%) in North America and 41 (18.4%) in Europe. Japan participated in 86 (38.6%) trials, ranking first globally, followed by China (51, 22.9%), the USA (43, 19.3%) and Germany (11, 5.0%).

For the sponsor type, there were 163 (45.4%) investigator-initiated clinical trial (IIT) and 37 (54.6%) industry-sponsored clinical trial (IST) studies, respectively. Japan initiated the most IIT studies (83/163, 50.9%), followed by China (43/163, 26.4%) and the USA (26/163,16.0%). The USA initiated a total of 10 (27.0%) IST studies, ranking first globally, followed by China (6/37, 16.2%) and Germany (4/37, 10.8%).

**Fig. 4:**
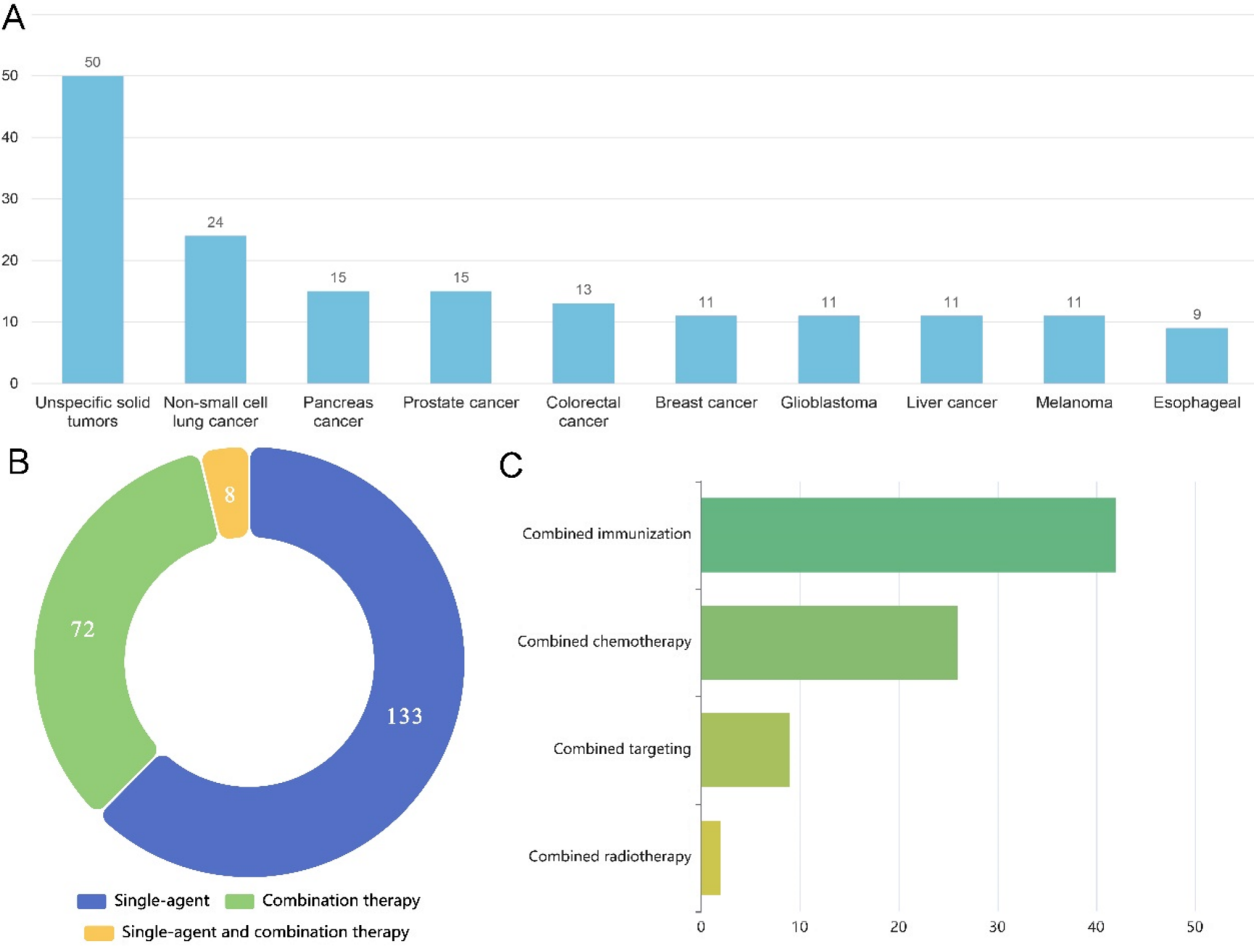
**A** Number of cancer types corresponding to the new antigen vaccine types used in registered clinical trials and during treatment. **B** Treatment modes and combination therapy of new antigen vaccines. **C** Treatment strategies for personalized neoantigen vaccines

The largest number of clinical trials was designed to treat three or more tumor types, here named unspecified solid tumor (50/199, 25.1%), followed by non-small cell lung cancer (24/199, 12.1%), and pancreas (15/199, 7.5%) (Fig. 4A). As multiple clinical trials included various types of cancers, each clinical trial was included in several categories of cancer types. From the perspective of treatment methods, monotherapy was mainstream (133/199, 66.8%), followed by combination therapy (72/199, 36.2%), with single agent and combination therapy being the least (8/199, 4.0%) (Fig. 4B). In our analysis, the specific contents of combination therapy were also provided to better understand the diversity of clinical trials for new antigen vaccines. Among them, 42 trials combined with immunotherapy, 26 combined with chemotherapy, 9 combined with targeted therapy, and 2 combined with radiotherapy (Fig. 4C).

Second and further lines accounted for the largest proportion of the distribution of treatment lines for all personalized neoantigen vaccines (119/199, 59.8%), with the smallest proportion used for first line (7/199, 3.5%). In addition, a high proportion of personalized neoantigen vaccines were observed for adjuvant/maintenance treatment (45/199, 22.6%), and further analysis of cancer types, combination treatments, and trial outcomes was performed. The top five cancer types in adjuvant/maintenance therapy were unspecified solid tumor (15/45, 33.3%), non-small cell lung cancer (7/45, 15.6%), colorectal cancer (4/45, 8.9%), breast cancer (4/45, 8.9%), and melanoma (4/45, 8.9%). In terms of treatment strategy for adjuvant/maintenance therapy, single agent remained the main modality (24/45, 53.3%) and combination therapy (18/45, 40%) was slightly higher than the overall 36.2% (Table 1).

**Table 1:** Distribution of cancer types by study phase of clinical trials for personalized neoantigen vaccines

Sponsor country	IIT	IST	Total
Japan	83	3	86
China	43	6	49
USA	26	10	36
Germany	2	4	6
Switzerland	2	2	4
UK	1	3	3
Netherland	1	1	2
Australia	1	0	1
Belarusian	1	0	1
Belgium	0	1	1
Canada	0	1	1
Egyptian	1	0	1
Singapore	0	1	1
South Korea	0	1	1
Sweden	0	1	1
Taiwan, China	1	0	1
Switzerland & Norway	0	1	1
Switzerland & Germany	0	1	1
Mexico	1	0	1
Total	163	37	199

**Fig. 5:**
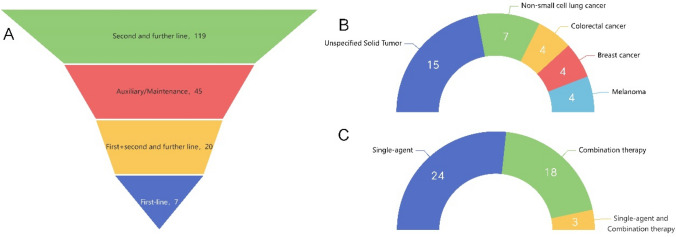
**A** Distribution of treatment lines for personalized neoantigen vaccines. **B** Top 5 cancer types targeted by adjuvant/maintenance therapy. **C** Treatment strategies for adjuvant/maintenance therapy

The detailed information on the results of clinical trials of personalized neoantigen vaccines for adjuvant/maintenance therapy is summarized in Table 2. As of early 2023, the preliminary results of 10 clinical trials were disclosed, including five for mRNA vaccines, four for peptide vaccines and one for DNA vaccines (Fig. 5).

**Table 2:** Results of preliminary clinical trials of personalized oncology neoantigen vaccines for auxiliary maintenance therapy

Protocol No	Types of vaccines	Delivery system	Indication	Sponsor country	Efficacy
NCT04147078	mRNA vaccine	LPX	Unspecified solid tumor	USA	At an early median follow-up of 15 months, vaccine responders (*n* = 8) had a longer RFS vs. non-responders (*n* = 8) (median not reached vs. 13.7 months, HR 0.08, 95% CI 0.01–0.5, *P* = 0.007) [15]
ChiCTR1900020990	Peptide vaccine	Peptide	Melanoma	China	Among 7 patients received all planned neoantigen vaccinations, 5 of them demonstrated neoantigen-induced T cell responses and have significantly longer RFS after radical surgery than other 5 patients without responsive neoantigens or only with prime vaccination and propensity scores matching control patients (*p* = 0.035) [16]
NCT03897881	mRNA vaccine	LNP	Melanoma	USA	18-month RFS rates (95% CI) were 78.6% (69.0%, 85.6%) vs 62.2% (46.9%, 74.3%) in the combination and monotherapy arm, respectively [17]
ChiCTR1800017319	mRNA vaccine	LPP	Unspecified solid tumor	China	NVAC induces both CD4 and CD8 T cell responses as well as antigen-experienced memory T cell phenotype. Furthermore, the immune response is persistent and remains evident one year after the vaccination [18]
NCT03548467	DNA vaccine	DNA	Non-small-cell lung	USA, Norway	T cell responses were elicited in both high and low tumor mutational burden patients (TMB range 2-69mut/Mb). Multiple vaccinations increased the breadth and magnitude of the immune responses with vaccine-induced T cell responses (de novo and/or amplified pre-existing) measured in 95% of eligible patients. [19]
NCT02808364	mRNA vaccine	DC	Acute lymphocytic leukemia	China	Among the seven patients tested for anti-TAA T cell responses, most of the TAAs induced antigen-specific CD4 and/or CD8 T cell responses, regardless of their expression levels in the tumor tissues [20]
NCT03559413	Peptide vaccine	Peptide	Chronic lymphocytic leukemia	Germany	Relapse incidence was low (22% at 3 years in all patients, 17% in CR1 patients) and was not impacted by any pre-transplant factors including positive MRD post phase 2 induction (present in 6 patients) [21]
NCT02721043	Peptide vaccine	Peptide	Glioblastoma	USA	Vaccine-specific T cell immunity was observed against multiple vaccine neoepitopes in all 13 subjects. Of the peptides administered, 45% of vaccine antigens (57/126) induced de novo immunity, starting as early as Week8 and often sustaining past last vaccination [22]
NCT03633110	Peptide vaccine	Peptide	Breast cancer	USA	Comparing RECIST responders (PR, CR) to non-responders (SD, PD), the median breadth of statistically positive responses to vaccine antigens at day 50 was greater in non-responders ex vivo (29 vs. 75%, respectively) [23]
NCT02316457	mRNA vaccine	LPX	Unspecified solid tumor	Germany	The highly poly-epitopic TCR-clonotype diversified CD8 T cell response comprised in aggregate about 30% of total peripheral CD8 T cells and was sustained at high levels for at least 6 months after the last vaccination. Vaccine-induced CD8 T cells were of effector/memory, PD1 phenotype, with a high fraction IFNγ/TNFα/Mip-1a&b triple-positive. [24]

## Discussion

This study presents a comprehensive overview of personalized neoantigen cancer vaccine clinical trials worldwide from 2003 to 2022. Clinical trials of all available neoantigen vaccines over this period are examined and analyzed in detail. The study shows the trends in vaccine types and delivery systems over time, demonstrates the types of cancer indications, and clarifies the use of treatment modalities and combination therapies. The objective is to provide a quantitative perspective on the diversity of categories, indications, and combinations in current clinical trials and to discuss the role of new antigen vaccines in cancer treatment, providing research data support for the industry, researchers, policy makers, and other stakeholders.

Immunotherapy, represented by immune checkpoint inhibitors (ICIs), has become an effective treatment for a variety of malignancies, effectively prolonging the survival of patients and reducing mortality, revolutionizing the cancer treatment. However, in some patients, it has shortcomings such as low response rates, a single target, significant side effects, and easy recurrence [25].On the one hand, the therapeutic effects of ICIs depend on the basic function of the body’s anti-tumor immune response [26], and tumor ICIs represented by PD-1/PD-L1 have limited response to "cold" tumor patients without immune cell infiltration in tumor tissues. On the other hand, there is a high heterogeneity in the gene mutations among individual tumor patients. Therefore, due to the clinical limitations of ICI therapy, regulating the host’s immune system has become a hot topic in immunotherapy research in recent years. Among them, tumor neoantigen vaccines induce the production of more tumor-specific T cells through antigen stimulation, reconstruct the anti-tumor immune system, and overcome the low immunity or immune incompetence induced during tumor development and progression. Preliminary efficacy has been observed in both animal models and clinical studies, and tumor neoantigen vaccines have a potential synergistic effect in combination with ICIs. Tumor neoantigen vaccines are expected to bring tumor immunotherapy to a new height, prolong patient survival, and benefit more patients.

Our results demonstrate that clinical development of cancer neoantigen vaccines has rapidly evolved after 2018. It is mainly attributed to the rapidly development of two technologies: neoantigen prediction tools, and vaccine delivery platform. Further progress in artificial neural networks and deep learning models are supporting to predict MHC/ligand interactions [27, 28]. Various formats of vaccine are being explored in clinical trials. The most frequency used vaccine formats are long peptides vaccine, with the mutated of delivery technologies, mRNA formulations, viral vectors and DNA with intrinsic adjuvant activity which encode a string of multiple predicted neoantigen are moved to clinical trial stage. Compared with peptide vaccines, new technology vaccines, especially mRNA vaccines with the liposomal platform can achieve individualized design, rapid production, thus reduce preparation time and financial costs [29, 30].

In our review, early data of neoantigen vaccines trials confirmed the acceptable safety profile and preliminary clinical efficacy (Table 2) as the later line treatment in advanced solid tumors. In specific tumor species, such as pancreatic cancer and malignant melanoma, tumor neoantigen vaccines achieve some clinical efficacy based on the understanding of tumor neoantigen and immunotherapy sensitivity. In previously reported clinical trials, the treatment strategies are tumor neoantigen vaccines in combination with immune checkpoint inhibitors. The sufficient immune effects cannot be activated by neoantigen vaccines alone, and combined immune checkpoint inhibitors are required to further activate T cell immunity [29, 31].

Tumor neoantigen vaccines present the loaded personalized antigens to the immune system and activate neoantigen-specific T cells to kill tumors. Like immune checkpoint inhibitors, its clinical efficacy is affected by factors such as tumor burden, mutation status, site of metastases, and immune microenvironment [32, 33]. Therefore, in the adjuvant therapy following surgery [12, 34] or maintenance therapy following chemotherapy [14] where the patients have less tumor burden, it is more suitable for tumor neoantigen vaccine to play a key role in removing minimal residual disease. At the 2023 AACR meeting, the results of a phase II study of mRNA-4157 combined with pembrolizumab showed that the combined regimen increased the 18-month RFS to 78.6% and reduced the risk of recurrence or death by 44% at 2 year follow-up compared with pembrolizumab in high-risk resected melanoma (HR = 0.561, *P* = 0.0266) [12]. This is the first proof of concept randomized study with positive results in this area. The pivotal study of mRNA-4157 combined with pembrolizumab in melanoma has been initiated in 2023, Jul (NCT05933577) and expected to be completed in 2029 [20].

Regardless of the rapid development of personalized cancer vaccine, several significant challenges due to the complexity and uniqueness of each patient’s cancer are being laid out in front of scientists.

1. Identifying neoantigens: neoantigens are mutated proteins expressed by cancer cells that can be targeted by the immune system. However, accurately identifying which neoantigens are present in a patient’s tumor requires advanced sequencing and bioinformatics analysis. This process must be both efficient and accurate to ensure the vaccine targets the most relevant antigens.
2. Manufacture: the productive process of tumor neoantigen vaccines is highly individualized and involves sequencing, neoantigen prediction, vaccine design, and production. How to maintain homogeneity and stability is a major challenge in the chemistry, manufacturing, and control (CMC) process [35, 36]. Due to the above features, the drug regulatory authorities have addressed high requirements for the production process of neoantigen vaccines applied for clinical trials as novel drugs. This challenge leads to the fact that the majority of global clinical trials were investigator initiated and only a few were industry sponsored, especially in Japan and China. On the contrary, the dual-track regulation system has also created more opportunities of early clinical explorations in these countries. Clinical experiences obtained from IITs may support the application for new drug registration trials by pharmaceutical enterprises [37]. As of the highly individualized characteristics, how to establish risk-based evaluation standards and fully integrate IIT and IST data requires in-depth consideration by the regulatory authorities.
3. Immunogenicity evaluation: knowledge gaps and shortage of experimental methods for quantitatively analyze the spatiotemporal changes of the immune system in patients after neoantigen vaccine administration are remain to be prominent., More basic clinical research paradigms are needed to elaborate the immunological mechanisms behind them. A variety of immunological assays have been applied in neoantigen vaccine clinical trials to analyze tumor-specific immunogenic responses after administration, including Elispot and peptide-MHC tetramer staining. Elispot is the most commonly used immunogenicity assay in neoantigen tumor vaccine trials, but with several limitations, as peripheral blood mononuclear cells need to be collected from patients and are greatly affected by operating procedures. How to develop a more objective quantitative analysis method to standardizes the operation and interpretation of Elispot is the next direction of experimental methodology for tumor neoantigen vaccine in the future. Peptide-MHC (pMHC) tetramers have been used to detect Ag-specific CD4 + and CD8 + T cells alone or combined with other flow cytometry panels. Technological challenges in pMHC terameters are how to improve detection sensitivity and anti-interference ability, such as the ability to recognize low-affinity TCRs, highly recognition rate of CD4 + T cells, and anti-interference power of anti-CD8 and anti-CD-4 antibodies [38].
4. Clinical efficacy evaluation: currently in cancer vaccines clinical trials after systemic therapy in advanced tumors or postoperative adjuvant therapy in early-stage patients, tumor responses of cancer vaccines currently have been evaluated utilizing traditional RECIST v1.1 criteria to assess efficacy. Per RECIST v1.1, in addition to enlargement of targeted tumor size, PD often being confirmed by the appearance of new lesions or unequivocal growth of nontarget lesions, However, patterns of response with immunotherapy, such as ICIs or cancer vaccines, differ from cytotoxic agents, with stable disease or PD being noted in some cases before response, such as pseudo progression (initial tumor growth followed by shrinkage). Mounting studies have reported that a proportion of patients treated with ICIs who are documented with PD per RECIST v1.1 actually have stable or reduced tumor burden. Therefore, developing and validating new response criteria that account for these patterns is crucial. In addition, the characteristic of efficacy induced by vaccines is delayed but durable tumor response, appropriate alternative clinical endpoints still needs to be explored, especially in trials to investigate neoantigen vaccines as later line treatment in advanced cancers.
5. Patient selection and stratification: identifying which patients are most likely to benefit from a personalized cancer vaccine is crucial. This involves developing biomarkers or other criteria to stratify patients based on their tumor biology, immune profile, and other factors.

Addressing these challenges requires collaboration among researchers, clinicians, regulatory agencies, and industry partners. Advances in technology, such as next-generation sequencing and computational biology, are helping to overcome some of these hurdles, but ongoing innovation and investment are needed to realize the full potential of personalized cancer vaccines in clinical practice.

## Conclusion

The clinical development of personalized neoantigen cancer vaccines is still in the early stage. A clear shift in delivery systems from peptides to DC and liposomal platforms, with the largest number of studies in Asia, collectively marks a new era in the field. The adjuvant or maintenance therapy, and the combination treatment with ICIs are becoming the important clinical development orientation. As research on tumor–immune interactions intensifies, the design, development and application of neoantigen vaccines are bound to develop rapidly, which will bring a new revolution in the future cancer treatment.

## Data Availability

The datasets generated and analyzed during the current study are available from the corresponding author on reasonable request.

## Abbreviations

TAAs: Tumor-associated antigens
TSAs: Tumor-specific antigens
ORFs: Open reading frames
MHC: Major histocompatibility complex
ICB: Immune checkpoint blockade
PD-1: Programmed cell death protein-1
TILs: Tumor infiltrating lymphocytes
TCR-T: T cell receptor engineered T cells
CAR-T: Chimeric antigen receptor T cells
DC: Dendritic cells
LNP: Lipid nanoparticle
LPX: Lipoplexes
LPP: Lipopolyplex
IST: Industry-sponsored clinical trial
IIT: Investigator-initiated clinical trial
